# A p53 Drug Response Signature Identifies Prognostic Genes in High-Risk Neuroblastoma

**DOI:** 10.1371/journal.pone.0079843

**Published:** 2013-11-19

**Authors:** Eveline Barbieri, Katleen De Preter, Mario Capasso, Peter Johansson, Tsz-Kwong Man, Zaowen Chen, Paris Stowers, Gian Paolo Tonini, Frank Speleman, Jason M. Shohet

**Affiliations:** 1 Texas Children’s Cancer Center and Center for Cell and Gene Therapy, Department of Pediatrics, Baylor College of Medicine, Houston, Texas, United States of America; 2 Center for Medical Genetics, Ghent University, Ghent, Belgium; 3 CEINGE Biotecnologie Avanzate, Department of Biochemistry and Medical Biotechnology, University of Naples Federico II, Naples, Italy; 4 Oncogenomics Section, Pediatric Oncology Branch, Center for Cancer Research, National Cancer Institute, National Institutes of Health, Bethesda, Maryland, United States of America; 5 Pediatric Research Institute, University of Padua, Padua, Italy; Institute of Cancer Research: Royal Cancer Hospital, United Kingdom

## Abstract

Chemotherapy induces apoptosis and tumor regression primarily through activation of p53-mediated transcription. Neuroblastoma is a p53 wild type malignancy at diagnosis and repression of p53 signaling plays an important role in its pathogenesis. Recently developed small molecule inhibitors of the MDM2-p53 interaction are able to overcome this repression and potently activate p53 dependent apoptosis in malignancies with intact p53 downstream signaling. We used the small molecule MDM2 inhibitor, Nutlin-3a, to determine the p53 drug response signature in neuroblastoma cells. In addition to p53 mediated apoptotic signatures, GSEA and pathway analysis identified a set of p53-repressed genes that were reciprocally over-expressed in neuroblastoma patients with the worst overall outcome in multiple clinical cohorts. Multifactorial regression analysis identified a subset of four genes (CHAF1A, RRM2, MCM3, and MCM6) whose expression together strongly predicted overall and event-free survival (p<0.0001). The expression of these four genes was then validated by quantitative PCR in a large independent clinical cohort. Our findings further support the concept that oncogene-driven transcriptional networks opposing p53 activation are essential for the aggressive behavior and poor response to therapy of high-risk neuroblastoma.

## Introduction

Neuroblastoma (NB), an embryonal tumor arising in tissues of the sympathetic nervous system, is the most common cancer diagnosed during the first year of life and accounts for 13% of all deaths due to childhood malignancies. Despite intense multimodality therapy, at least half of high-risk patients will experience relapse that is almost always fatal [1]. Much of the difficulty in devising effective therapies for this latter group of patients lies in the heterogeneity of their disease, which can be attributed largely to the interaction of multiple genetic factors, including both sequence and copy number variants [2], [3]. Amplification of the *MYCN* gene has emerged as one of the most reliable indicators of aggressive and treatment-resistant neuroblastoma, yet 30% to 40% of high-risk tumors lack this feature [1]. Genomic studies, including massively parallel DNA sequencing, have failed to reveal additional recurrent molecular lesions in neuroblastoma, with the exception of ALK mutations found in a small percentage of high-risk tumors [4], [5], [6].

A number of previous observations confirm that wild-type p53 alleles are present in the vast majority of cases of newly diagnosed neuroblastoma, but that p53/MDM2/ARF responses to chemotherapy are repressed, in part due to unscheduled inhibition of p53 by MDM2 [7], [8], [9]. This suggests that down- regulation of the p53 axis may underlie the treatment resistance typically seen in high-risk neuroblastoma. To further investigate downstream p53-effector genes contributing to this cancer phenotype, we used Nutlin-3a, an MDM2 antagonist, which specifically activates p53 leading to apoptosis and tumor regression of both chemosensitive and chemoresistant neuroblastoma cell lines [10], [11], [12]. We compared gene expression profiles before and after Nutlin treatment and used bioinformatic approaches to identify p53 drug response genes whose aberrant expression in high-risk disease may limit p53 activation in response to genotoxic chemotherapy, increasing the risk of disease progression and relapse. With this approach we identified four genes that are highly over expressed in high-risk neuroblastoma (CHAF1A, RRM2, MCM3, and MCM6) whose expression strongly correlates with poor outcomes. Importantly, these genes are all directly repressed by Nutlin-3a-induced p53 activation, are markers of aggressive disease in other cancers, and have functions related to tumorigenesis and aberrant DNA replication and transcription [13], [14], [15], [16].

## Methods

### Tissue Culture

JF (ATCC), IMR32 (ATCC), LAN5 (LS Metelitsa, Houston TX), and LAN1 (ATCC) human NB lines were maintained in RPMI 1640; human colorectal cancer cell line, HCT 116 (ATCC), and human breast cancer cell line, MCF7 (ATCC), in McCoy’s 5A and DMEM plus 1% insulin respectively; human osteosarcoma line, SJSA-1 (ATCC), in RPMI 1640 medium with 10 mM HEPES, 1 mM sodium pyruvate, 4.5 g/L glucose, 1.5 g/L sodium bicarbonate; primary neuroblastoma lines (p202, p218, pH) (Texas Children’s Cancer Center, Houston, TX) and CHLA255 line (LS Metelitsa) in IMDM with 20% FBS and 0.1% ITS. The p53-mutant NB line, SJ3-12 (21 amino acids deletion in the DNA binding domain), was provided by Dr. Dirk Geerts, University of Amsterdam, the Netherlands and used as control [9]. Nutlin-3a (provided by Dr. Vassilev - Roche, Nutley, NJ) was used at the concentration of 10 uM (in DMSO).

### Oligonucleotide Microarray Data Analysis

Total RNA was isolated using RNAeasy kit (Quiagen) from the early passage cell line p202 at the 3, 8 and 16 hrs time points. Using Affymetrix™ U133a microarrays, we compared expression profiles of Nutlin-3a and Nutlin-3b (inactive enantiomer) treated p202 primary NB cells over time points. Two biological replicates were carried out for time-series experiments. Microarray data were analyzed by an ANOVA time series model using FDR < 0.1 as a statistical cutoff. Class and time comparison analysis identified probe sets differentially expressed between Nutlin treatments (class effect) and time points (time effect). Data were clustered using Cluster 3.0, with centered correlation as distance measure for both genes and arrays.

### Gene Set Enrichment Analysis (GSEA)

For each time point, genes were ranked with respect to the average expression change upon Nutlin-3a treatment. GSEA was then performed for each of the three time points using gene permutation alternative. Enrichment analysis was done with default parameter settings. An enrichment score was calculated for each gene set (KS-statistics) reflecting if the genes in the particular gene set appeared in the top (positive score), in the bottom (negative score), or were randomly distributed (close to zero score). These scores were compared with scores calculated from 1,000 randomly permuted gene lists, in order to calculate false discovery rates (FDR) (cutoff at FDR = 0.05) [17].

### Clinical Patient Cohort Groups

1. Oberthuer set (n =  251), discovery set 1. 251 NB tumors profiled on custom Agilent 44k arrays and downloaded from ArrayExpress EBI (http://www.ebi.ac.uk/arrayexpress) (E-TABM-38) [18].

2. Neuroblastoma Research Consortium (NRC) set (n =  101), discovery set 2. 101 NB tumors profiled on the Human Exon 1.0 ST Affymetrix array [19].

3. Wang set (n =  99), discovery set 3. 99 NB tumors profiled on Affymetrix U95Av2 array (Gene Expression Omnibus -GEO- database, http://www.ncbi.nlm.nih.gov/geo/) [20].

4. Versteeg set (n =  88), discovery set 4. 88 NB tumors profiled on the Affymetrix HGU133plus2.0 platform (R2 database, microarray analysis and visualization platform, http://r2.amc.nl).

5. Khan set (n =  56), discovery set 5. 56 primary NB tumors (Oncogenomics, http://home.ccr.cancer.gov/oncology/oncogenomics) [21].

6. Vermeulen set (n = 348), discovery set 6. 348 NB tumors taken from the International Society of Pediatric Oncology, European Neuroblastoma Group (SIOPEN) and from the Gesellschaft fuer Paediatrische Onkologie und Haematologie (GPOH). Patients were only included if primary untreated tumor RNA samples were available and of sufficient quality [22]. Expression of the four gene signature was evaluated in this cohort using real-time quantitative PCR.

### Data Analysis and Statistics of Gene Expression in Neuroblastoma Tumors

Gene expression data were evaluated according to methods previously developed for assessment of gene/survival outcomes [19], [22]. Signature scores of the 25- or 4-gene lists were obtained by calculating the sum of the ranks of the (standardized) expression values of the 25 or 4 genes across the different samples in a dataset. Expression of the gene set correlates with signature score [23]. Alternatively, we performed Prediction Analysis for Microarrays (PAM) classification using the MCR estimate R package. Kaplan-Meier survival analysis and log-rank analysis were carried out using the R survival package (R version 10.1). Multivariate logistic regression analyses were done using SPSS (version 16). Currently used risk factors such as age at diagnosis (≥12 months vs. <12 months), International Neuroblastoma Staging System (INSS) stage (stage 4 vs. other stages), and *MYCN* status (amplified vs. non-amplified) were tested. GEMS algorithm [24] was used to construct support vector machine (SVM) predictors using 20-fold cross-validation for each clinical factor: death event (DE), relapse event (RE), and INSS stage. The efficiency of the predictor was estimated by a receiver operator characteristic (ROC) analysis and recorded as the area under the curve (AUC).

### Real-Time qPCR

A qPCR assay was designed for each of the four genes and five reference genes by PrimerDesign and went through an extensive in silico validated analysis using BLAST and BiSearch specificity, amplicon secondary structure, SNP presence, and splice variant analysis. The mean amplification efficiency was 98%. Primer design and real time quantitative PCR analysis were performed as described [22]: primer sequences are available in RTPrimerDB [25]: CHAF1A (ID = 8273), RRM2 (ID = 8270), MCM6 (ID = 8271), MCM3 (ID = 8272) and reference genes: HPRT1 (ID = 5), SDHA (ID = 7), UBC (ID = 8), and HMBS (ID = 4). Data handling and calculations (normalization, rescaling, inter-run calibration, and error propagation) were done in qBasePlus version 1·1 (http://www.qbaseplus.com) [26] [27].

### Plasmids and Antibodies

ShRNA mediated p53 knockdown: To knock down p53 expression, second generation lentiviruses expressing shp53 and shLuc control were used as described [28]. Briefly, 293T cells were transfected with pLSLPw construct along with packaging plasmids, pVSVG and pLV-CMV-delta 8.2 by using lipofectamine. Virus-containing supernatants were collected at 48 and 72 hours and neuroblastoma cells transduced in the presence of 8 mg/ml polybrene (Sigma).

## Results

### Transcriptional profiling of p53 response in neuroblastoma identifies important genes with respect to outcome

**Figure 1:**
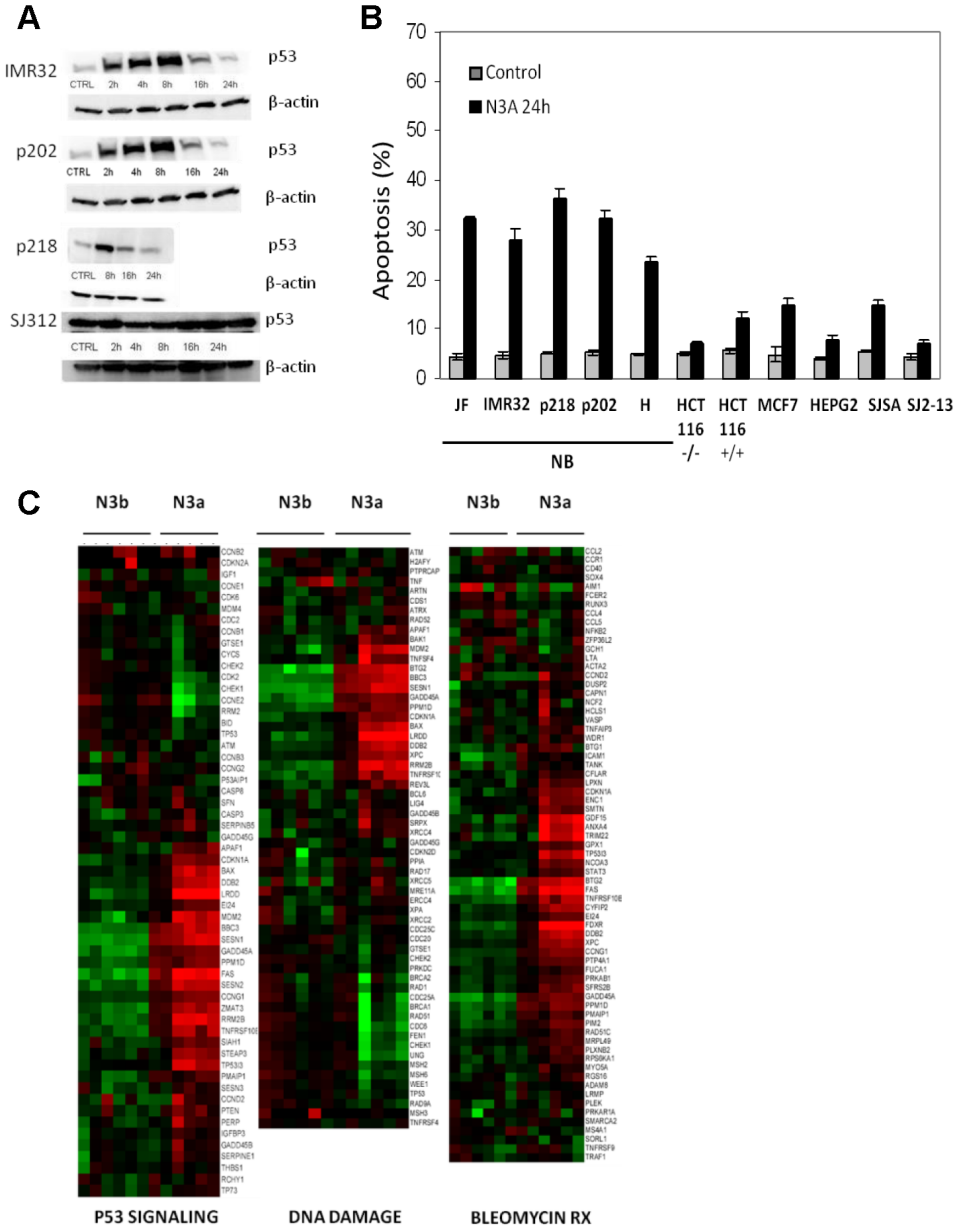
MDM2 inhibition stabilizes p53 and induces apoptosis and p53 signaling pathways in neuroblastoma cells. (A) Consistent with our previously data [11], Western blotting showed an increase in p53 levels within 4 hours exposure to Nutlin-3a in p202, p218, and IMR32 cells, confirming that the p53 signaling pathway is active in our system. (B) Apoptosis induction was tested in five established neuroblastoma lines (JF, IMR32), early passage neuroblastoma tumor cultures (p202, p218, H), and four non-neuroblastoma human solid tumors including colorectal (HCT116), breast (MCF7) and osteosarcoma (SJSA-1). SJSA-1 line differs by the level of MDM2 expression, being amplified 25 fold. A p53 mutant neuroblastoma line (SJ3-12), which lacks part of the DNA binding domain, was used as control. Proliferating cells were treated with Nutlin-3a for 24 hours and TdT-positive fraction was measured by flow cytometry. (C) Pathways enriched upon Nutlin-3a treatment: p53 signaling (GSEA ID: M6370), DNA damage (GSEA ID: M8378), and chemotherapy response (bleo_human_lymph) genes. Vertical columns separate Nutlin-3a versus Nutlin-3b at 3, 8, and 16 hours, horizontal rows indicate genes.

To initially assess the p53-dependent response we used the MDM2 inhibitor Nutlin-3a to stabilize p53 in a panel of primary (p202, p218, and H) and established neuroblastoma lines and tested its ability to induce apoptosis in neuroblastoma cells compared to other solid tumors. At 24 hours all neuroblastoma lines showed a higher percentage of apoptosis compared to non-neuroblastoma lines. Consistent with our previously published data [11], Western blot analysis showed a very rapid increase in p53 levels within four hours exposure to Nutlin-3a, confirming that the p53 signaling pathway is active in our lines **(** **Figure 1A and 1B** **)**. Interestingly, p53 levels return to baseline at later time points, likely due to robust MDM2-mediated ubiquitination and degradation in these cell lines. We then compared the expression profiles of a *MYCN* amplified primary neuroblastoma line (p202) treated with Nutlin-3a or its inactive enantiomer Nutlin-3b for 3, 8, and 16 hours. As expected for a p53 wild-type tumor, we identified a large number of genes regulated by p53: 1285 genes differentially expressed between the two Nutlin treatments (class effect) and 201 genes differentially expressed between time points (time effect) **(Table S1 and Table S2)**.

We then used Gene Set Enrichment Analysis (GSEA) to analyze genes and cellular pathways mainly affected by p53 activation. For each time point genes were ranked with respect to the average change in expression. Based on these ranked gene lists GSEA was performed [17]. As expected, three gene sets, p53 signaling (GSEA ID: M6370), DNA repair (GSEA ID: M8378), and chemotherapy response (bleo_human_lymph) were highly up-regulated due to over expression of known p53 target genes **(** **Figure 1C** **)**.

**Figure 2:**
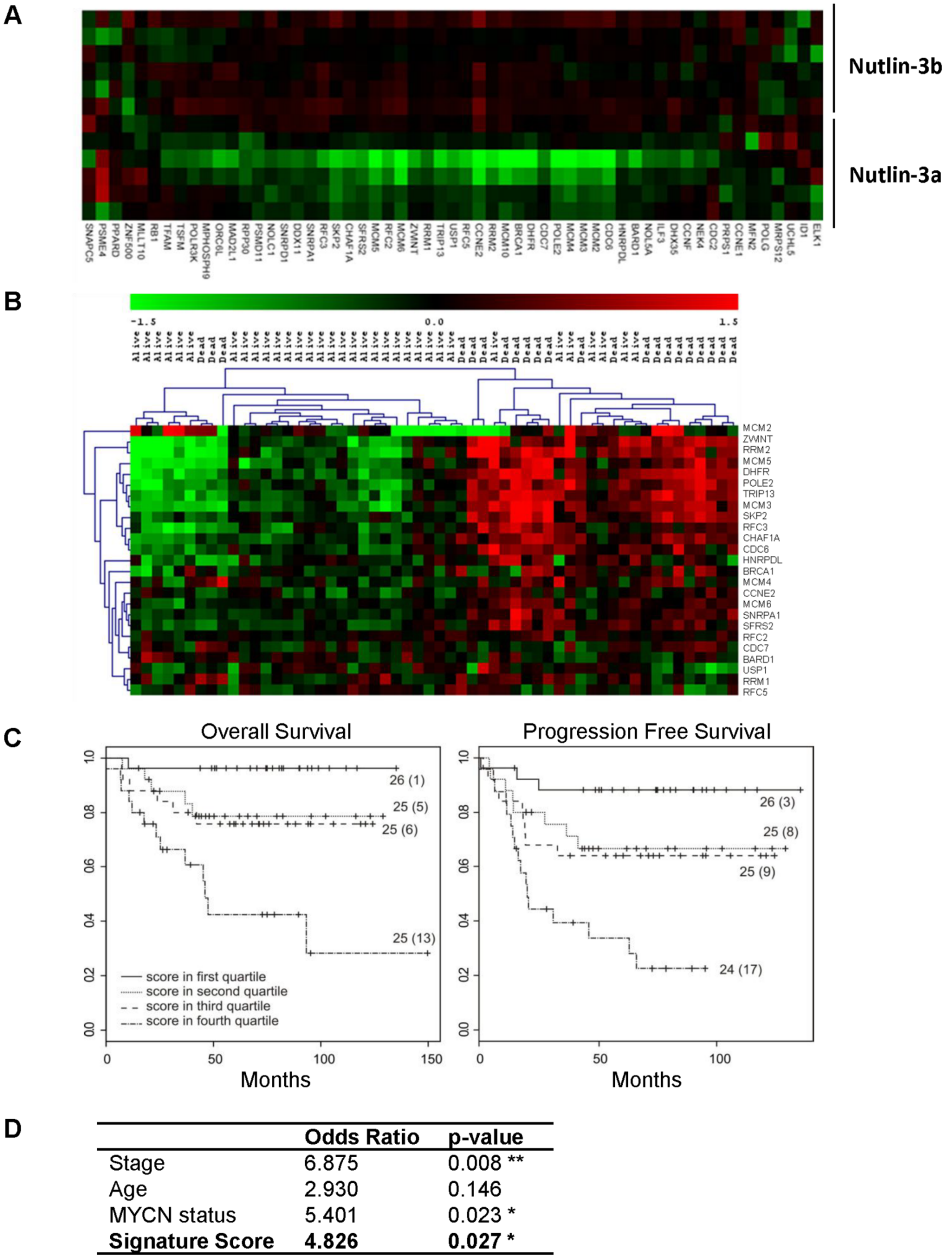
Correlation of p53 repressed genes and poor prognosis. (A) Heat map of the gene set significantly down-regulated by Nutlin-3a but not Nutlin-3b. (B) Heat map with hierarchical clustering of the gene set with respect to neuroblastoma prognosis (discovery set 5). (C) Kaplan–Meier and log-rank analysis for progression-free (PFS) and overall (OS) survival of discovery set 2 based on 25 gene set: survival of 101 neuroblastoma patients in the 4 quartiles of the signature score. Numbers in parentheses refer to number of patients who experienced an event. (D) Multivariate logistic regression analysis: Odds Ratio and p-values are shown for disease stage (stage 4 vs. other), age (< or > 12 months), *MYCN* status (amplified vs. non-amplified), and the 25-gene signature score according to methods previously published [19].

By contrast, a fourth gene set was down-regulated upon p53 activation in neuroblastoma cells. This set (GSEA ID: M18562) included 57 unique genes described to be repressed both by HIF1A and hypoxia in endothelial cells (p<0.0001) [29] **(** **Figure 2A** **)**. p53 has long been shown to play key roles in responding to DNA damage, hypoxia, and oncogenic activation. Hypoxia is also known to modulate the p53 transcriptional activity in a manner dependent and independent of HIF1A, the main transcription factor activated by hypoxia [30]. As repression of p53 functions is critical to neuroblastoma tumorigenesis, we hypothesized that these p53-repressed genes may be important in neuroblastoma biology and asked whether they could define patient outcome. We found that high expression of a subset of this gene set (25 genes) correlated with poor survival in a cohort of 56 neuroblastoma tumor samples (discovery set 5) [21] **(** **Figure 2B** **and Table S3)**.

We next investigated the prognostic value of this subset of 25 genes in an independent set of 101 neuroblastoma samples (discovery set 2) by calculating signature scores as previously described [19]. Increased expression of this gene set significantly correlated with poor outcome by log-rank analysis (overall survival (OS) p = 0.00018; progression-free survival (PFS) p = 0.00013) **(** **Figure 2C** **)**. In addition, multivariate analysis incorporating signature score, *MYCN* status, stage and age demonstrated that samples with high expression of this gene set had five times higher predicted and independent mortality than samples with low expression (odds ratio: 4.826; p = 0.026) **(** **Figure 2D** **)**. The predictive power of this gene set was then independently validated in three additional clinical cohorts of high-risk patients with Kaplan-Meier and log-rank analysis (discovery set 1 [18] - 251 patients: OS p<0.00001, PFS p<0.00001), discovery set 3 [20] - 99 patients: OS p<0.05, PFS p<0.005), and discovery set 4 - 88 patients: OS p<0.005 PFS, p<0.01) **(Figure S1)**.

### Identification of a four-gene signature with significant prognostic value in neuroblastoma patients

**Figure 3:**
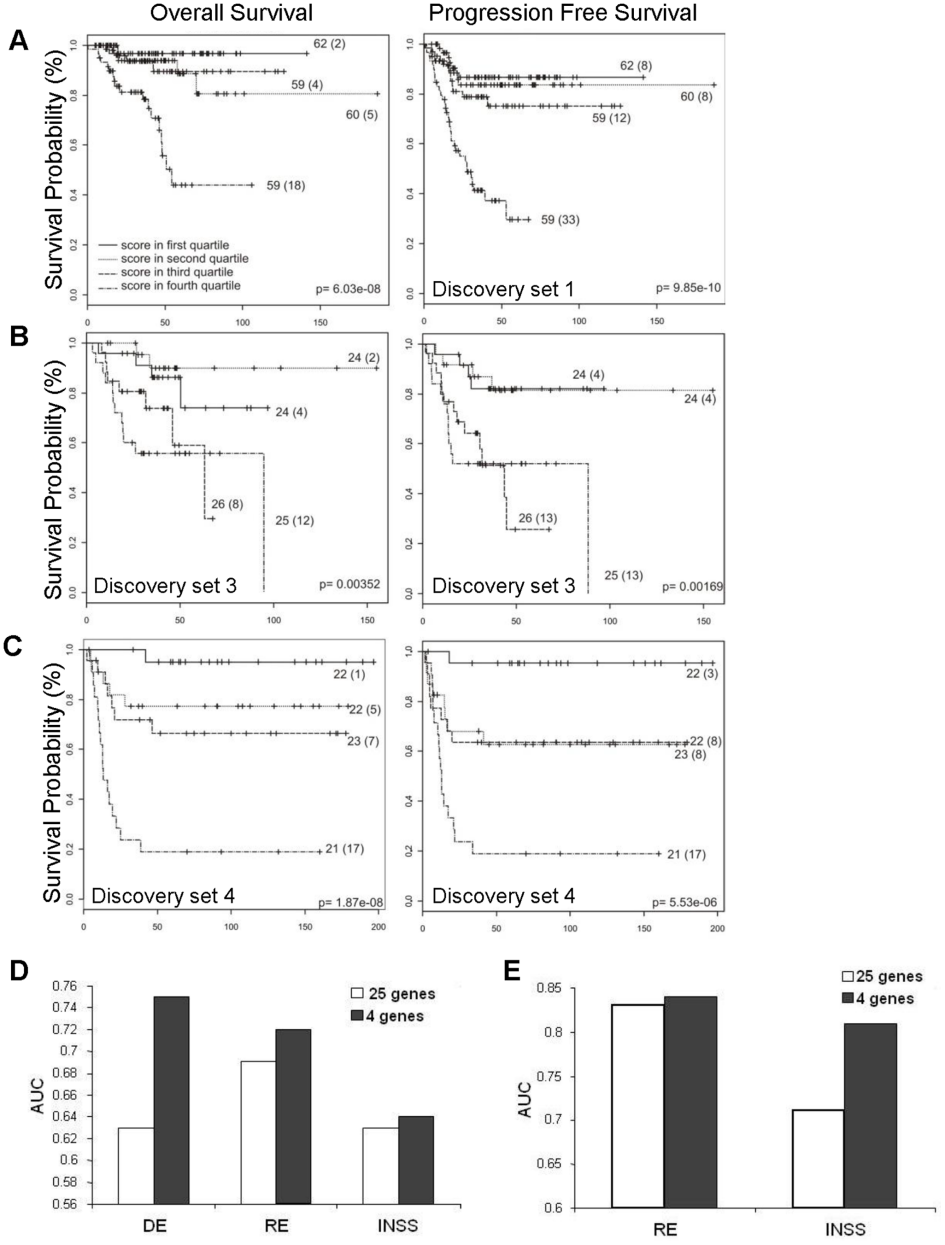
The four-gene signature predict neuroblastoma outcome. Kaplan–Meier and log-rank analysis for progression-free (PFS) and overall (OS) survival in discovery set 1 (A), set 3 (B), and set 4 (C) based on the four-gene signature score. (D, E) Comparison of signature performance between all 25 and the 4 selected genes. Results from discovery and validation study strategy. (D) Gene predictors made in discovery set 4 and predictions made in discovery set 1. (E) Gene predictors made in validation set 4 and predictions made in discovery set 5. X axis indicates clinical factors: death event (DE), relapse event (RE), and INSS stage; Y axis indicates Area Under Curve (AUC).

We predicted that a subset of these 25 genes reflected a fundamental aspect of the p53-mediated response, and therefore we sought to define which p53 repressed genes mostly contributed to the prognostic power of this signature. Thus, a multivariate logistic regression analysis of 101 patients of the discovery set 2 (with at least 36 months follow up) was performed as described in the Methods section. This analysis revealed a subset of four genes (CHAF1A, RRM2, MCM3, and MCM6) as independent predictor of overall survival (p<0.1). The correlation of survival with signature score based on the expression of these four genes was then validated in three independent data sets (discovery set 1- 251 patients: OS p<0.00001, PFS p<0.00001, discovery set 3 - 99 patients: OS p<0.005, PFS p<0.005, and discovery set 4 - 88 patients: OS p<0.00001, PFS p<0.0005) (**Figure 3A, 3B and 3C**).

We also applied an alternative method based on a discovery and validation strategy to assess the power of the four genes to predict mortality, relapse, and INSS stage. A classifier able to predict these clinical factors was independently generated by the SVM (Support Vector Machine) algorithm (discovery set 4, n = 88 patients) [24]. The classifier was then validated in two independent patient cohorts (discovery set 1 - 251 patients and discovery set 5 – 56 patients). ROC-curve analysis (AUC) shows that the classifier containing only the four genes performs better than the classifier containing all 25 genes in predicting mortality, relapse, and INSS stage **(** **Figure 3D and 3E** **).**

### Independent validation of the four-gene signature by quantitative-PCR in a large clinical cohort

**Figure 4:**
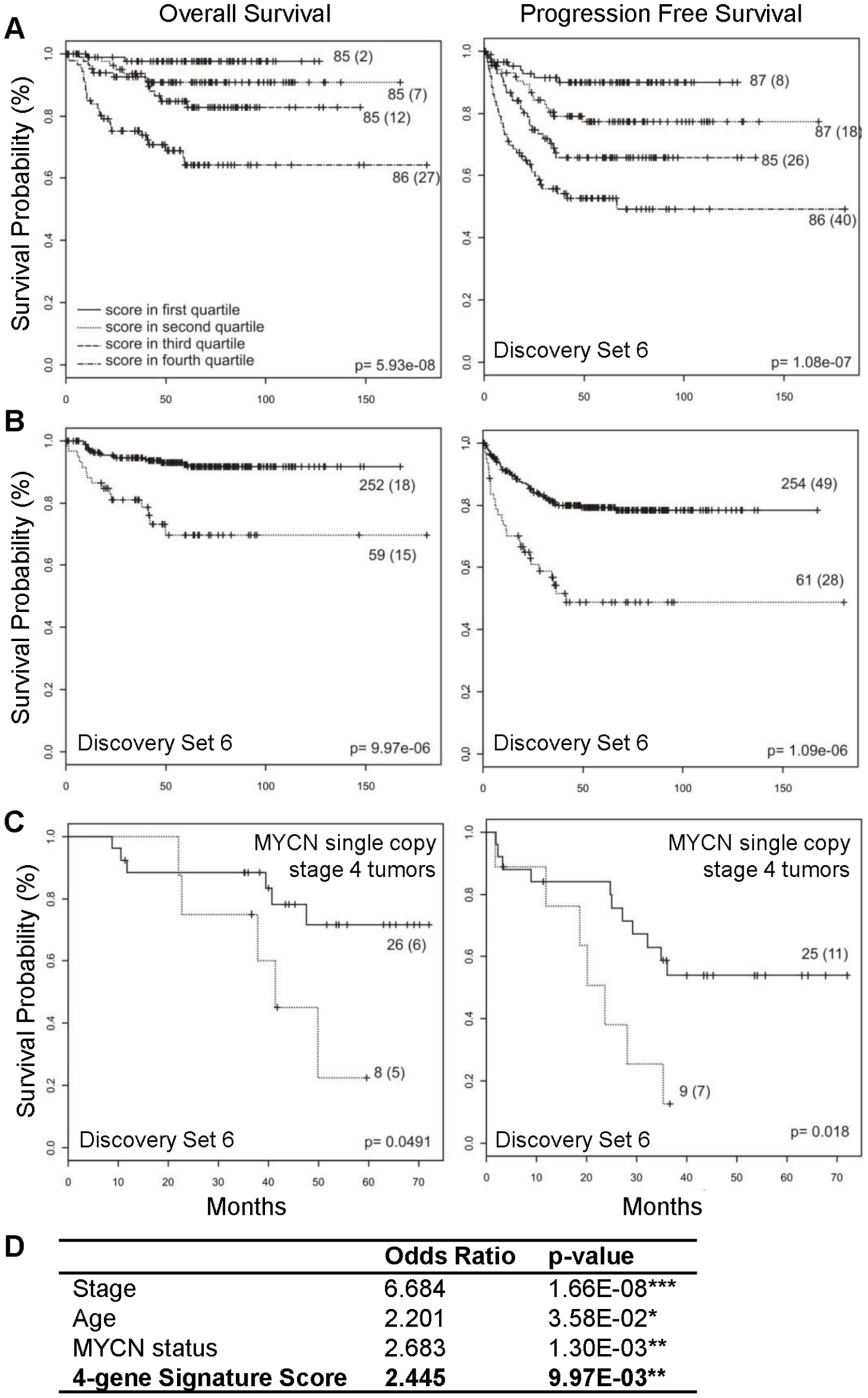
Independent validation of the four-gene signature in a large cohort of neuroblastoma patients (SIOPEN/GPOH). (A) Kaplan–Meier and log-rank analysis for progression-free (PFS) and overall (OS) survival of the SIOPEN/GPOH cohort (discovery set 6): survival of 348 neuroblastoma patients in the 4 quartiles of the signature score. (B) Validation of the signature based on the four-gene PAM classifier. (C) Classification of the SIOPEN stage 4 tumors with *MYCN* single copy based on the four-gene PAM classifier. (D) Multivariate logistic regression analysis: Odds Ratio and p-values are shown for disease stage (stage 4 vs. other), age (<> 1y), *MYCN* status, and the four-gene signature score.

Extending these findings to an additional large independent clinical cohort of neuroblastoma patients, we performed qPCR to evaluate gene expression of CHAF1A, RRM2, MCM3 and MCM6 in tumor samples from 384 patients enrolled in the International Society of Paediatric Oncology Europe Neuroblastoma Group (SIOPEN) and the Gesellschaft fuer Paediatrische Onkologie und Haematologie (GPOH) clinical trials (validation set 6) [22]. Both signature score and PAM classification show that the expression of these four genes as determined by qRT-PCR distinguishes patients with respect to progression-free (p<0·0001) and overall survival (p<0·0001) (**Figure 4A and 4B**). Progression-free survival at five years from the date of diagnosis was 0.793 (95% CI 0.742 – 0.847) for the group of patients at low molecular risk compared with 0.487 (95% CI 0.366– 0.649) for the group of patients at high molecular risk. The five year overall survival was 0.916 (95% CI: 0.878–0.955) and 0.697 (95% CI 0.574 –0.847) in the low and high molecular risk groups, respectively.

Currently, the strongest predictive risk factors used for neuroblastoma risk stratification are age, stage, tumor histology, and *MYCN* gene amplification status. Multivariate logistic regression analysis revealed that high expression of the four genes predicts poor survival independently of these clinical features (odds ratio: 2.4; p<0.01) **(** **Figure 4D** **)**. To validate that the four-gene signature was independent of the major risk factor, *MYCN* status, we analyzed only the *MYCN* single copy stage 4 tumors. Indeed, the signature can efficiently predict survival in this group of patients lacking *MYCN* gene amplification (log-rank analysis, p< 0.05) **(** **Figure 4C** **)**.

### The four-gene signature is repressed by p53 in neuroblastoma cells

**Figure 5:**
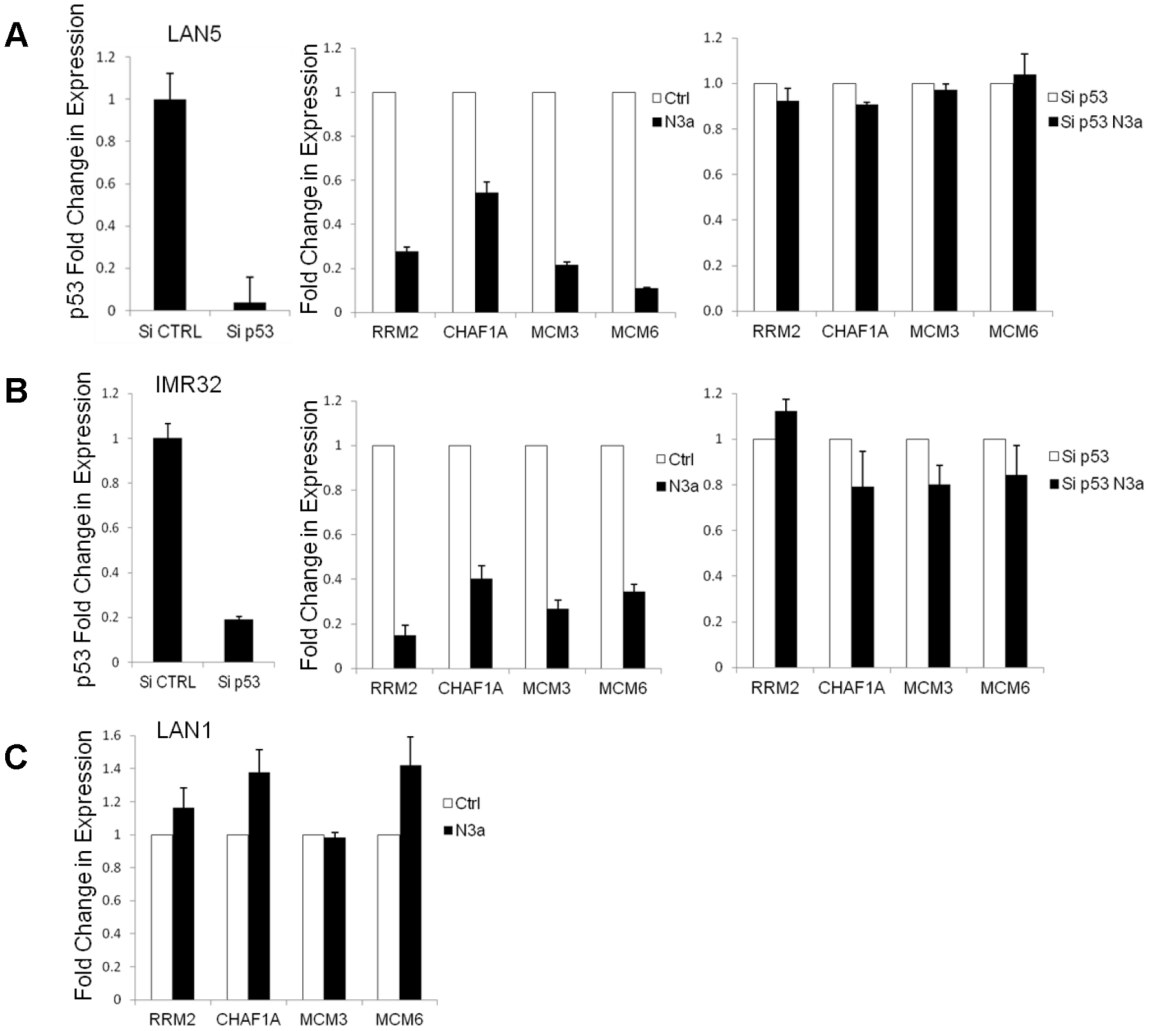
The four-gene signature is regulated by p53 activity. Activation of p53 represses the four-gene signature. (A, B) Quantitative PCR demonstrates marked decrease of mRNA levels of the four genes after Nutlin-3a treatment (10 uM for 8 hours) in multiple p53 wild-type neuroblastoma lines (shown here LAN5 and IMR32). However, this effect is completely abrogated after p53 silencing or when the effect of Nutlin-3a is tested in a p53 mutant neuroblastoma cell line (LAN1) (C). Each error bar represents two biological replicates.

To test that the expression of these four genes is indeed regulated by p53, gene expression of RRM2, CHAF1A, MCM3, and MCM6 was assessed in multiple p53 wild-type neuroblastoma lines treated with Nutlin-3a. Real-time PCR demonstrated robust and rapid repression of each of these genes upon treatment (p<0.005). However, this effect was totally abrogated in neuroblastoma p53 mutant (LAN1) cell line which lacks a DNA binding domain. In addition, we used ShRNA-mediated knockdown of p53 in several cell lines and confirmed up-regulation of all four target genes **(** **Figure 5A, 5B and 5C** **).** Furthermore, analysis of two publically available independent ChIP-seq data sets [31], [32] confirms clear p53 binding to p53 response elements in the promoters of these four genes in response to p53 activation (data not shown). Taken together, these data suggest that these genes are direct p53 transcriptional targets.

## Discussion

As noted above, p53 is rarely mutated in neuroblastoma, suggesting that repression of downstream effector genes is critical for tumorigenesis. p53 is a potent transcription factor that positively and negatively modulates a large number of genes involved in apoptosis, metabolism, epigenetics, and cell cycle regulation [33], [34]. We used Nutlin-3a, a direct inhibitor of the p53 E-3 ligase MDM2, to investigate how the p53 drug response signature is regulated in high-risk neuroblastoma. Starting from the analysis of a gene set repressed by p53 activity and highly expressed in high-risk neuroblastoma, we identified a novel four-gene signature of prognostic relevance. In our retrospective analysis of three large independent cohorts of neuroblastoma patients (540 patients total) and 348 patients enrolled in the SIOPEN/GPOH, this four-gene signature classifies patients with respect to survival independently of current clinical classification scheme based on age, stage, *MYCN* expression and histological features (INRG Staging System [3]). Notably, our four-gene signature shows predictive value in neuroblastoma patients with stage 4 disease and without *MYCN* amplification. As over 50% of these patients will relapse and succumb to neuroblastoma despite current risk stratifications, there is clearly a need for additional identification of higher risk subgroups that could be given alternative or additional therapies.

The four genes constituting the signature are involved in DNA replication and chromatin remodeling. In addition, along with other genes involved in cell growth, they have been described as repressed by HIF1A and hypoxia in endothelial cells [29]. It is well known that tumor microenvironment is intimately connected with neuroblastoma biology [35], [36], [37]. While the influence of HIF1A on the expression of these four genes in neuroblastoma remains to be defined, we demonstrated that their expression strongly correlates with poor outcome.

These four genes are likely to have roles in determining neuroblastoma behavior. RRM2, MCM3 and MCM6 genes have all been described as cell cycle regulatory genes repressed by p53 in other models as well [13], [38]. Ribonucleotide Reductase M2 subunit (RRM2), which is expressed when DNA replication occurs, is overexpressed in a number of solid tumors and is an established anti-cancer target [15]. Additional evidence suggests that Ribonucleotide Reductase (RR) acts as a positive determinant for tumor cell proliferation and metastasis as well as the development of chemoresistance [39]. p53 and RRM2 also directly interact to regulate DNA damage responses [38]. A recent phase I clinical trial involving the administration of RRM2 siRNA via nanoparticle to patients with solid cancers showed promising results [40]. In addition, several inhibitors (like triapine and GTI-2040) have been studied as potential inhibitors of RRM2 activity and have been evaluated in phase I clinical trials [41]. A recent study suggests that RRM2 contributes to the stabilization of Bcl-2, a marker of chemoresistance [42]. BCL2 levels are high in the majority of neuroblastoma tumors. These observations suggest that suppression of Bcl-2 by targeting RRM2 may be an effective strategy for restoring chemo-sensitivity in neuroblastoma.

Growing data support the importance of perturbations of the DNA replication machinery in driving cancer. The minichromosome maintenance (MCM) gene family is essential for DNA replication and is frequently upregulated in various cancers. Furthermore, increased expression of MCM genes has been shown to correlate with poorer patient survival rates in other tumor types [43], [44], [45]. Notably, all the MCM genes, including MCM7, are direct transcriptional targets of *MYCN* and E2F [16], [46]. In addition, deregulation of G1-S checkpoint and E2F target genes correlates with poor outcome in neuroblastoma [47], further supporting a potential oncogenic role of MCM genes in neuroblastoma.

The histone chaperone molecule, CHAF1A (CAF p150), is a primary component of the chromatin assembly factor 1 (CAF-1) which is vital for DNA assembly during S-phase [14], [48]. CHAF1A inactivates cell proliferation, regulates DNA repair, and controls epigenetic marking in embryonic stem cells [49]. CHAF1A is also a known epigenetic modifier. By participating in a complex with MBD1 and SETDB1, CHAF1A promotes H3K9 trimethylation and heterochromatin formation [48], [50]. Importantly, CHAF1A overexpression has been linked to tumor progression, genomic instability, and cancer susceptibility in other tumor types including glioma [51], [52], [53].

The tumor suppressor p53 is very rarely mutated in primary neuroblastoma at diagnosis and its downstream effectors are functionally intact [8], [54]. However, multiple hits seem to cooperate to impair p53 functions in neuroblastoma, including deregulation of the ARF/MDM2 pathway [55], expression of microRNAs targeting p53 pathways [56], and repression of p53-mediated autophagy [57]. We demonstrate here that transcriptional profiles of high-risk neuroblastoma show *over- expression* of genes that are *repressed* by p53, and furthermore, a subset of these genes has strong prognostic value, suggesting they are involved in overcoming chemotherapy-induced p53 activity. The therapeutic approach of ‘reactivating p53’ (e.g. by MDM2 antagonists, or delivery of wild type p53) for neuroblastoma, which develops in the context of *MYCN*-mediated p53 repression is an active area of investigation with several early phase clinical trials underway [58], [59]. Our data further support these approaches and suggest additional targets for incorporation into future translational studies.

## Supporting Information

Figure S1 **Kaplan-Meier and log-rank analysis for progression free and overall survival of three independent cohorts of NB patients stratified according the expression of the 25 genes.** Survival of 251 patients of discovery set 1 (A), 99 patients of discovery set 3 (B), and 88 patients of discovery set 4 (C) in the 4 quartiles of the signature score. Represented is the number of patients at low and high-risk as predicted by the 25-gene signature. Numbers in parentheses refer to number of patients who experienced an event.
(TIFF)

Table S1 **Selected genes modulated by Nutlin-3a treatment.** Genes differentially expressed between Nutlin-3a and Nutlin-3b treatment (class effect – Table S1) and between time points (time effect – Table S2) are listed.
(XLSX)

Table S2 **Selected genes modulated by Nutlin-3a treatment.** Genes differentially expressed between Nutlin-3a and Nutlin-3b treatment (class effect – Table S1) and between time points (time effect – Table S2) are listed.
(XLSX)

Table S3 **Correlation of the 25 genes with survival in discovery set 5.** The list of the 25 genes repressed by p53 and the correlation of their expression with stage of disease in 56 neuroblastoma patients are shown.
(XLS)
