# Supplementary figures and images for: A p53 Drug Response Signature Identifies Prognostic Genes in High-Risk Neuroblastoma
Source: PLoS One. 2013 Nov 19;8(11):e79843. doi: 10.1371/journal.pone.0079843 (PMC3865347; doi:10.1371/journal.pone.0079843)

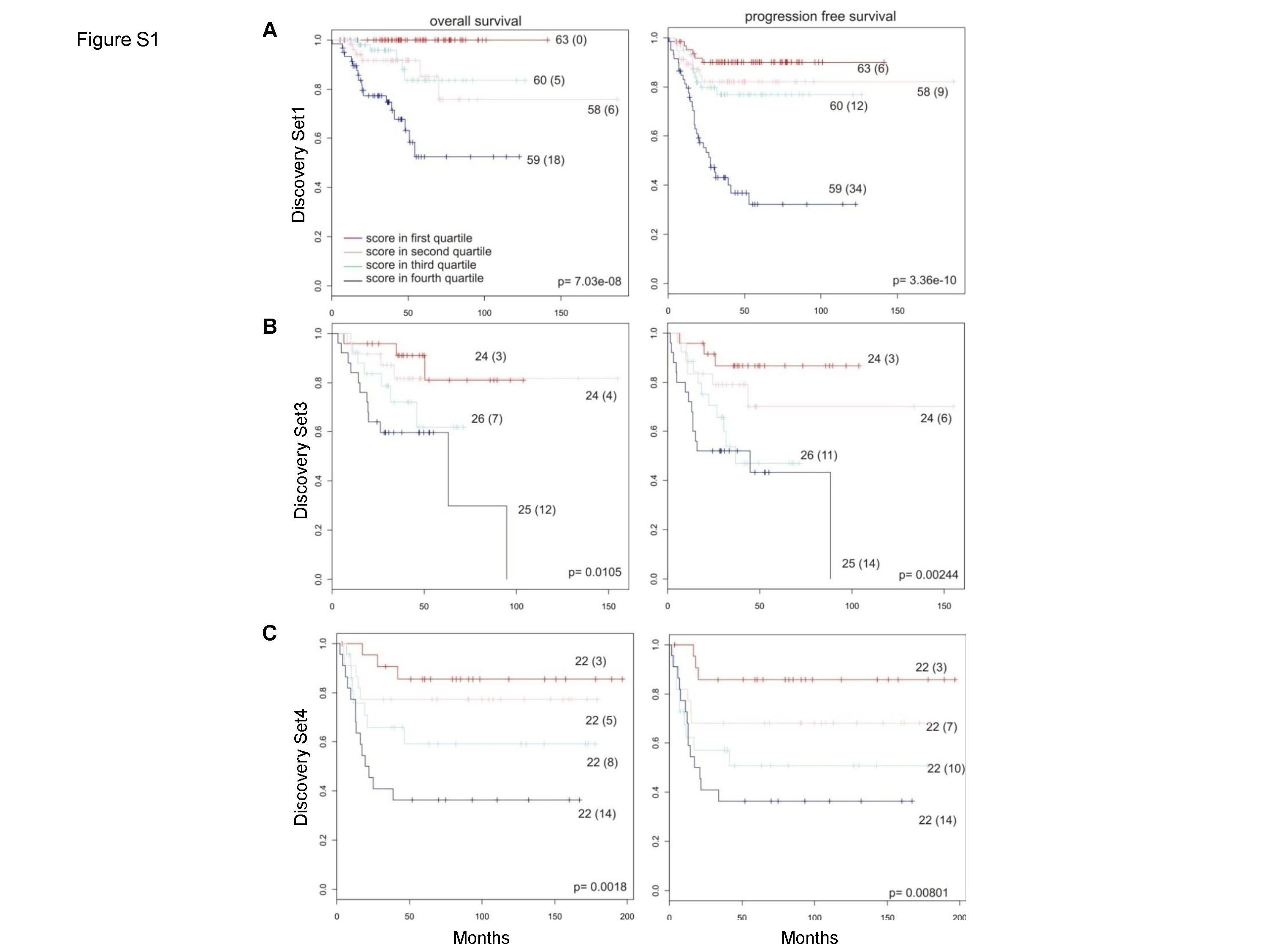

Supplement: Figure S1 — Kaplan-Meier and log-rank analysis for progression free and overall survival of three independent cohorts of NB patients stratified according the expression of the 25 genes. Survival of 251 patients of discovery set 1 (A), 99 patients of discovery set 3 (B), and 88 patients of discovery set 4 (C) in the 4 quartiles of the signature score. Represented is the number of patients at low and high-risk as predicted by the 25-gene signature. Numbers in parentheses refer to number of patients who experienced an event. (TIFF) [file pone.0079843.s001.tiff]
